# National Institutes of Health–Funded Artificial Intelligence and Machine Learning Research, 2019‐2023: Cross-Sectional Study

**DOI:** 10.2196/84861

**Published:** 2026-01-08

**Authors:** Joshua Pei Le, Joseph Morrison, Atul Malhotra, Shamim Nemati, Gabriel Wardi, James S Ford

**Affiliations:** 1 School of Medicine, University of Limerick, Limerick, Ireland; 2 School of Medicine, University of California Davis, Sacramento, CA, United States; 3 Department of Medicine, University of California San Diego, La Jolla, CA, United States; 4 Department of Emergency Medicine, University of California San Diego, 9500 Gilman Drive, La Jolla, CA, 92093, United States, 1-404-518-8923

**Keywords:** artificial intelligence, AI, machine learning, ML, National Institutes of Health, NIH, research funding

## Abstract

Inflation-adjusted funding for artificial intelligence and machine learning research increased by 233% between fiscal year 2019 and 2023, outpacing the overall National Institutes of Health’s budget increase of 12%.

## Introduction

Artificial intelligence (AI) and machine learning (ML) technologies have begun to revolutionize the practice of medicine. Recent studies have shown that 2 in 3 physicians currently use AI in their clinical practice and more than 3 in 4 scientists currently use AI in their research [1]. Recognizing the growing importance of AI tools, the National Institutes of Health (NIH) began tracking AI and ML as its own funding category in fiscal year (FY) 2019. While previous studies have broadly assessed NIH funding trends for AI and ML research, no studies report data more recently than 2020 [2,3]. Moreover, little is known about the principal investigators (PIs) driving this research. In this study, we aimed to examine trends in NIH-funded AI and ML research and characterize the population of funded PIs.

## Methods

We conducted a cross-sectional study of NIH RePORTER (Research Portfolio Online Reporting Tools Expenditures and Results) data between October 1, 2018, and September 30, 2023. We extracted studies indexed under the “Machine Learning and Artificial Intelligence” funding category and collected data on funding institute, mechanism, and award amount (in US $). We also selected a random sample of approximately 25% of PIs who were awarded grants, and we conducted an internet search to collect data on educational background (eg, MD), research setting (eg, academic), and clinical specialty (as appropriate). We searched official websites (eg, university), LinkedIn, or publicly available curriculum vitae. To account for potential differences in educational background (eg, PhD) and clinical specialty (eg, internal medicine) among PIs funded by different institutes, we balanced our random sampling according to the original institute-level proportions of funded grants. Three authors (JM, JPL, and JSF) conducted the search. CIs for proportions were calculated using the Wald method. Tests of trend were conducted using Poisson and linear regression, as appropriate. Analyses were performed using Stata/SE (version 17.0; StataCorp LLC).

## Results

**Figure 1.**
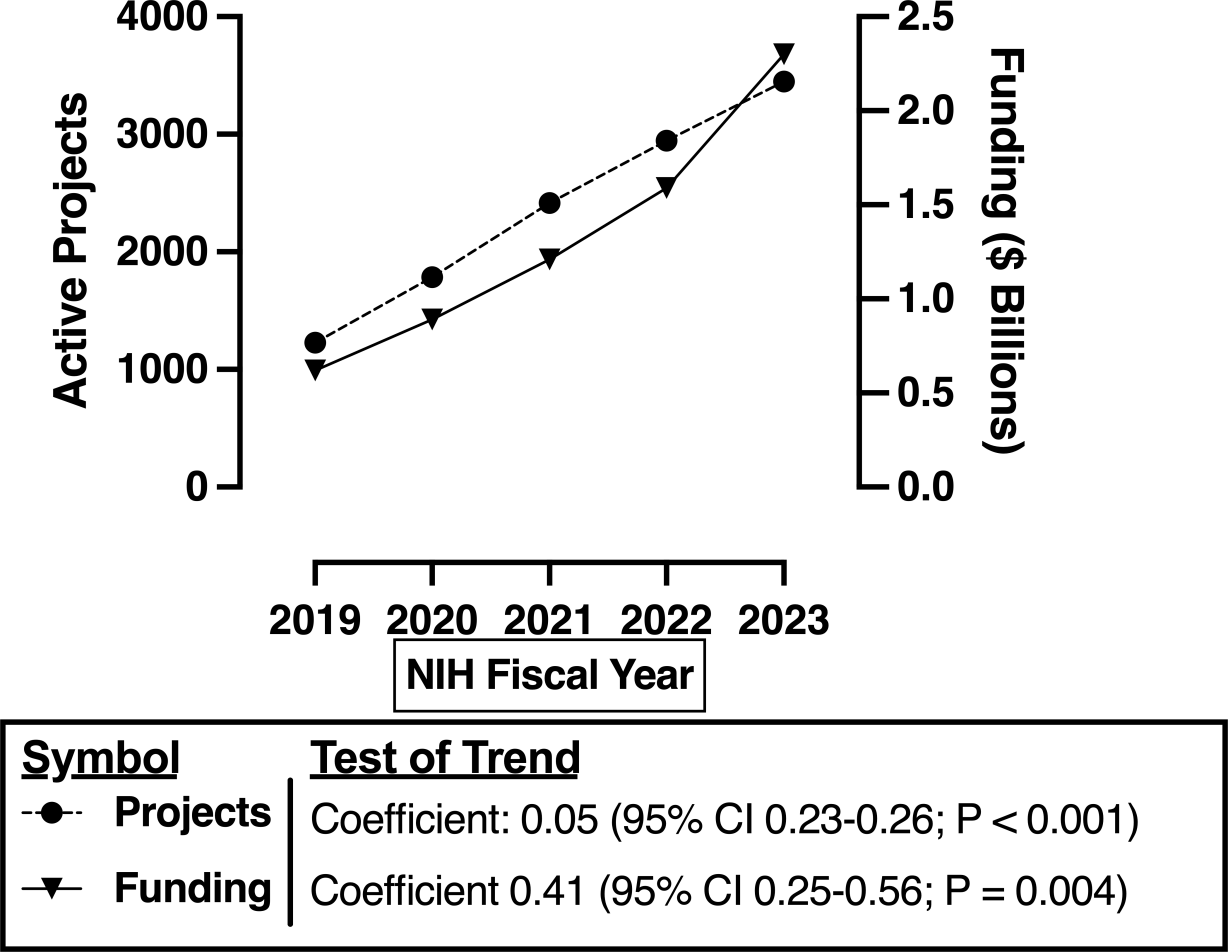
Overall National Institutes of Health (NIH)–funded artificial intelligence and machine learning research projects and funding (in US $) between NIH fiscal year 2019 and 2023. Plotted funding (US $) represents nominal values unadjusted for inflation. Inflation-adjusted values are presented in Table S2A and S2B in Multimedia Appendix 1.

Total active projects (including multiyear grants) increased from 1229 to 3449 and total funding increased nominally from US $0.6 billion to $2.3 billion between NIH FY 2019 and 2023 (Figure 1).

**Figure 2.**
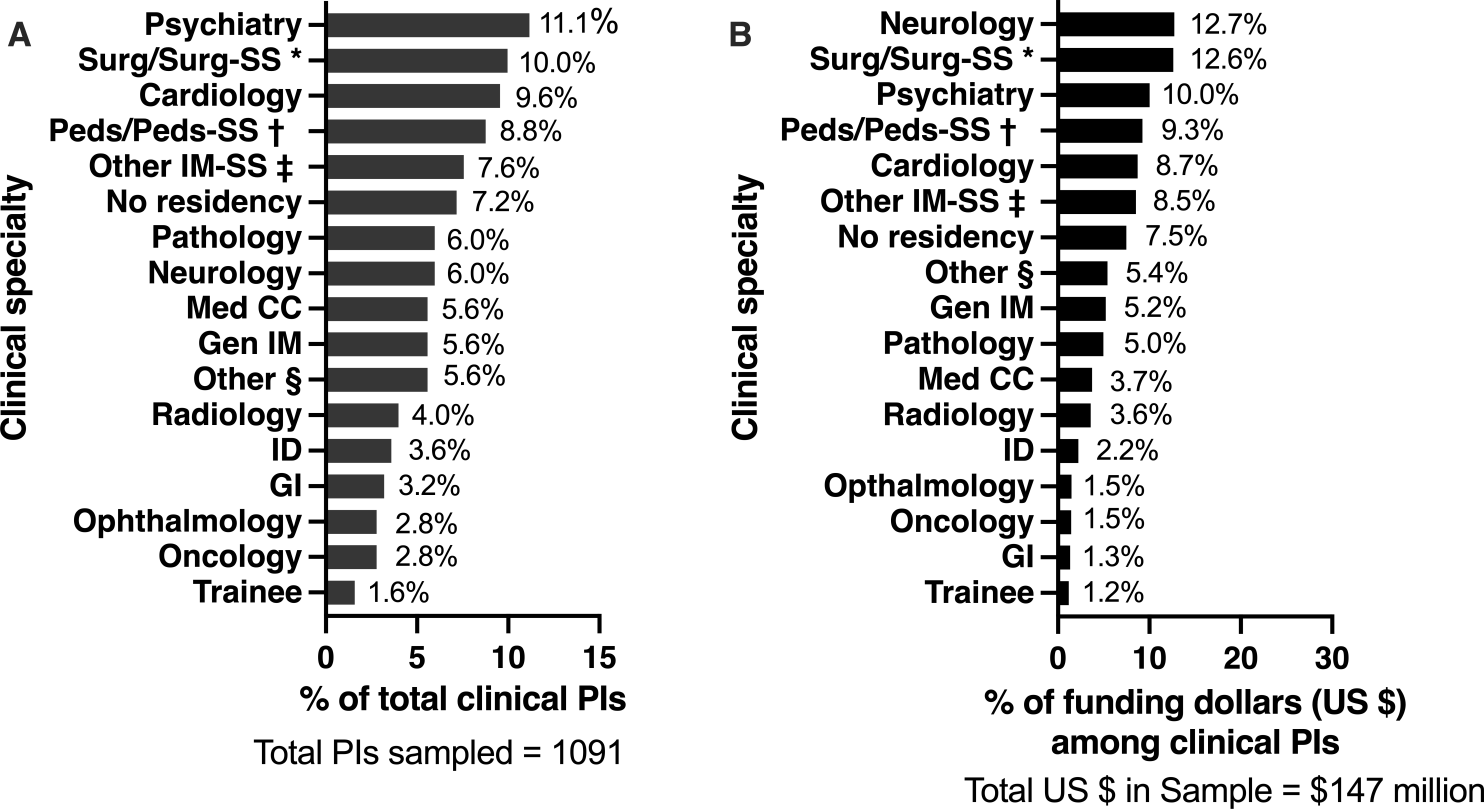
(A) Proportions of total funded principal investigators (PIs) and (B) total funding by specialty. Data from random sample of 1091 unique PIs with National Institutes of Health–funded projects in artificial intelligence or machine learning. CC: critical care; Gen: general; GI: gastroenterology; ID: infectious diseases; IM: internal medicine; Med: medical; Peds: pediatrics; SS, subspecialty; Surg: surgery. *Includes general surgery, urology, vascular surgery, transplant surgery, head and neck surgery, pediatric surgery, neurosurgery, obstetrics and gynecology. †Includes general pediatrics, cardiology, infectious diseases, hematology/oncology, pulmonology, endocrinology, neonatology. ‡Includes endocrinology, rheumatology, nephrology, hematology/oncology. §Includes emergency medicine, anesthesiology, pediatric emergency medicine, dermatology, and PIs with more than one specialty.

There were 5418 unique projects from 4365 unique researchers. Funding trends according to NIH institute and grant mechanism are available in Figures S1 and S2 in Multimedia Appendix 1. We randomly selected approximately 25% of PIs (1091/4395) to conduct further data extraction via internet searches. Among this random sample, the most common educational background was PhD only (70%, 758/1091), followed by MD or DO only (11%, 115/1091), and MD or PhD (9%, 93/1091) (Table S1 in Multimedia Appendix 1). Individuals with only a PhD accounted for 64% of funding dollars, compared to 18% of those with a medical doctoral degree (eg, MD or DO) but no PhD, and 11% for those with a clinical or professional degree (RN, MD, or PharmD) and a PhD. There were 251/1091 (23%) PIs who held medical doctoral degrees (MD or DO), and of these, 229 (91%, 95% CI 87%-94%) listed residency training. The proportions of total projects and funding dollars by medical specialty are available in Figure 2.

## Discussion

In 2017, which was before the NIH began tracking AI and ML as its own spending category, one study found that the NIH supported 535 projects totaling US $264 million, representing approximately 0.7% of the total NIH budget (NIH budget FY 2017; $34.301 billion) [2,4]. Our analysis of 2023 NIH RePORTER funding revealed $2.3 billion in funding (including direct, indirect, and supplemental costs) in this spending category, representing approximately 4.7% of the total NIH budget (NIH budget FY 2023; $49.2 billion) [4]. While the NIH budget increased by 12% (adjusted for inflation) between 2019 and 2023, funding for AI and ML research increased by 233% (adjusted for inflation) over this same period (Table S2A and S2B in Multimedia Appendix 1) [4].

Most AI and ML researchers held only a PhD (70%), and this group accounted for almost two-thirds of all NIH research funding dollars in the AI and ML funding category. By contrast, researchers who held a medical doctoral degree (MD or DO) and who had completed a clinical residency program were relatively underrepresented, accounting for less than 20% of funded researchers and total research dollars. In our sample of researchers who held a medical doctoral degree (MD or DO), those with training in psychiatry had the most funded projects and accounted for the third-highest proportion of funding dollars. While the list of possible applications is extensive and growing, notably, funding among specialties such as emergency medicine, anesthesiology, dermatology, ophthalmology, and various individual medical or surgical subspecialties was considerably lower. These gaps are similar to those seen across other research disciplines and represent clear targets for future NIH strategic investment [5,6].

As US federal funding priorities have been modified by the current administration and the NIH faces anticipated contraction in federal discretionary spending for FY 2026, our findings come at a critical moment [4,7]. Indeed, a recent study demonstrated the potential “ripple effects” of NIH funding cuts, including delays in innovation, workforce attrition, loss of talent to universities and corporations overseas, and increased long-term health care costs [8]. These proposed changes are likely to substantially weaken the United States’ contributions to this critical emerging field.

Our analysis included PIs, but data on coinvestigators were not captured. Data related to PI education and training background were obtained from publicly available online sources, which may have been incomplete or outdated at the time of collection. PIs with multiple specialties may have been misclassified. Our spending estimates are higher than those published by the NIH because we aggregated all funding (eg, direct costs, indirect costs, subprojects, and supplemental costs); we believe this methodology provides a more complete view of total research funding [9]. While our random sample of PIs was balanced according to institute-specific proportions, we cannot exclude the possibility of sampling bias. Lastly, the study period overlaps with the COVID-19 pandemic, which may confound funding trends during this time.

In summary, we found that total NIH projects and funding dollars increased substantially between 2019 and 2023. As the US government has enacted substantial NIH budget cuts for FY 2026, future studies are needed to examine the impact of current fiscal policy on future NIH-funded AI and ML research.

## Supplementary material

10.2196/84861 Multimedia Appendix 1 Supplemental Tables S1 and S2 and Figures S1 and S2.

## Abbreviations

AI: artificial intelligence
FY: fiscal year
ML: machine learning
NIH: National Institutes of Health
PI: principal investigator
RePORTER: Research Portfolio Online Reporting Tools Expenditures and Results
