# Supplementary material for: National Institutes of Health–Funded Artificial Intelligence and Machine Learning Research, 2019‐2023: Cross-Sectional Study
Source: J Med Internet Res. 2026 Jan 8;28:e84861. doi: 10.2196/84861 (PMC12782054; doi:10.2196/84861)
Supplement: Multimedia Appendix 1 [file jmir-v28-e84861-s001.docx]

**SUPPLEMENT**

| **Table S1.** Characteristics of randomly selected 25% sample of NIH-funded ML/AI principal investigators with projects indexed in NIH RePORTER, Fiscal Years 2019-2023 | | |
| --- | --- | --- |
| **Characteristics of Sampled PIs (n=1,091)** | **n (%)** | **95% CI** |
| **Research Setting (n=1,089)^1^** |  |  |
| Academics | 840 (77%) | 75% - 80% |
| Industry | 131 (12%) | 10% - 14% |
| Government | 87 (8%) | 6% - 10% |
| Independent Research Center | 31 (3%) | 2% - 4% |
| **Education (n=1,081)^2^** |  |  |
| PhD-Only | 758 (70%) | 63% - 73% |
| MD/DO-Only | 115 (11%) | 9% - 13% |
| MD, PhD | 93 (9%) | 7% - 10% |
| MD, Masters | 43 (4%) | 3% - 5% |
| Doctoral Student | 22 (2%) | 1% - 3% |
| Masters-Only | 22 (2%) | 1% - 3% |
| PhD, Other * | 20 (2%) | 1% - 3% |
| Bachelors-Only | 7 (1%) | 0.2% - 1.3% |
| PharmD Only | 1 (0.1%) | 0.002% - 0.514% |
| NIH, National Institutes of Health. PI, principal investigator.  ^1^Data unavailable for 2 PIs.  ^2^Data unavailable for 10 PIs.  * Includes: PhD, RN; PhD, RDN; PhD, DVM; PhD, OD; PhD, PharmD; PhD, DPT; DDS, PhD; PhD, RD; PhD, CRNA; PhD, MSW | | |

**Table S2A & S2B**

**S2A. NIH Spending on “Machine Learning & Artificial Intelligence”**

| Fiscal Year | Nominal ($ Billion) | Inflation Adjusted ($ Billion)* |
| --- | --- | --- |
| 2019 | 0.62 | 0.69 |
| 2020 | 0.89 | 0.99 |
| 2021 | 1.21 | 1.31 |
| 2022 | 1.59 | 1.65 |
| 2023 | 2.3 | 2.3 |

Percent Increase 2019 to 2023 = 233%

**S2B. Total NIH Spending**

| Fiscal Year | Nominal ($ Billion) | Inflation Adjusted ($ Billion)* |
| --- | --- | --- |
| 2019 | 39.31 | 43.76 |
| 2020 | 41.69 | 46.40 |
| 2021 | 42.94 | 46.63 |
| 2022 | 46.18 | 47.94 |
| 2023 | 49.18 | 49.18 |

Percent Increase 2019 to 2023 = 12.4%

*We have adjusted all dollars to constant 2023 dollars using the NIH Biomedical Research and Development Price Index (BRDPI).

The relative conversions to standardize to 2023 Dollar values are listed below^1^:

2019: 1.113

2020: 1.113

2021: 1.086

2022: 1.038

**^1^Source**: Department of Health & Human Services. “Biomedical Research and Development Price Index: Fiscal Year 2023 Update and Projections for FY 2024-FY 2029.” Accessed: October 27, 2025. URL: officeofbudget.od.nih.gov/pdfs/FY24/GDP/BRDPI%20Proj%20Memo%20-%20Final%20(January%202024).pdf

**
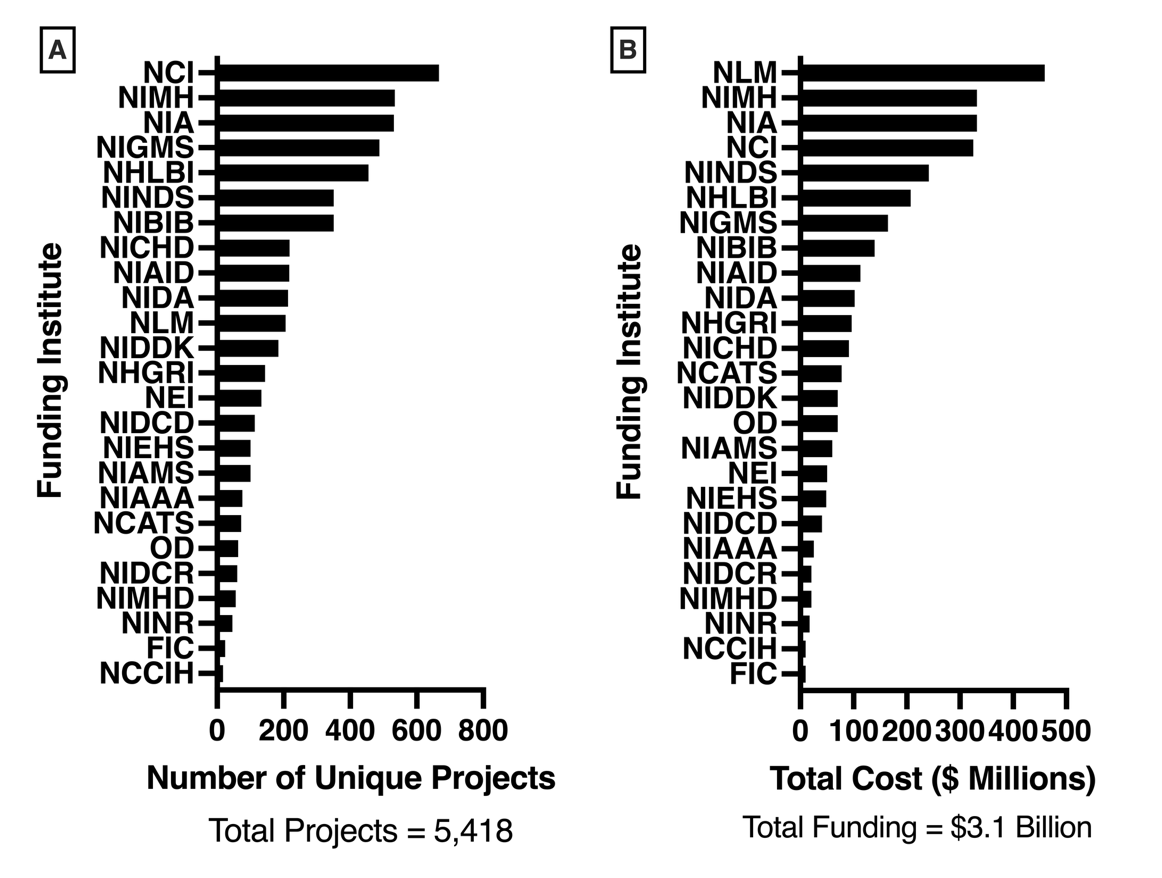
**

**Figure S1.** Funding for machine learning and artificial intelligence research by project count (S1A) and total cost (Figure S1B), according to NIH Institute.

NCI, National Cancer Institute; NIMH, National Institute of Mental Health; NIA, National Institute on Aging; NIGMS, National Institute of General Medical Sciences; NHLBI, National Heart, Lung, and Blood Institute; NIBIB, National Institute of Biomedical Imaging and Bioengineering; NINDS, National Institute of Neurological Disorders and Stroke; NICHD, Eunice Kennedy Shriver National Institute of Child Health and Human Development; NIAID, National Institute of Allergy and Infectious Diseases; NIDA, National Institute on Drug Abuse; NLM, National Library of Medicine; NIDDK, National Institute of Diabetes and Digestive and Kidney Diseases; NHGRI, National Human Genome Research Institute; NEI, National Eye Institute; NIDCD, National Institute on Deafness and Other Communication Disorders; NIAMS, National Institute of Arthritis and Musculoskeletal and Skin Diseases; NIEHS, National Institute of Environmental Health Sciences; NIAAA, National Institute on Alcohol Abuse and Alcoholism; NCATS, National Center for Advancing Translational Sciences; OD, Office of the Director; NIDCR, National Institute of Dental and Craniofacial Research; NIMHD, National Institute on Minority Health and Health Disparities; NINR, National Institute of Nursing Research; FIC, Fogarty International Center; NCCIH, National Center for Complementary and Integrative Health.

**
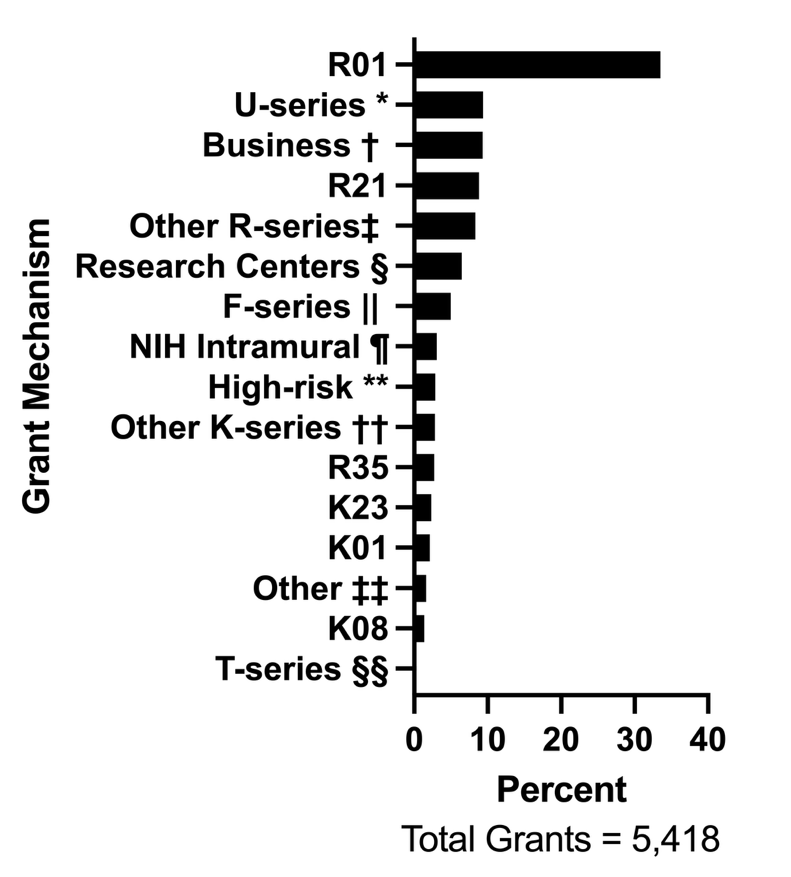
**

**Figure S2.** Common grant mechanisms for funded projects.

^*^ Includes: U01, U02, U13, U18, U19, U2C, U2R, U24, U34, U44, U54, UF1, UG1, UG3, UH2, UH3, UL1, UM1, UE5, UC2

† Includes: OT2, OT3, R41, R42, R43, R44, SB1

‡ Includes: **R00, R03, R13, R15, R16, R18, R24, R25, R33, R34, R36, R37, R50, R56, R61, R90**

§ Includes: **P01, P2C, P20, P30, P41, P42, P50, P60**

|| Includes: **F30, F31, F32, F99**

¶ Includes: **ZIA, ZIC, ZID, ZIG, ZIH, ZII, ZIJ**

** Includes: **DP1, DP2, DP3, DP5, RF1, RM1**

†† Includes: **K00, K02, K07, K12, K18, K22, K24, K25, K38, K43, K76, K99, KL2**

‡‡ Includes: D43, S06, S10, SC2, SC3, “Special Contracts”

§§ Includes: **T15, T32, T34, T35, T90, TL1**
